# Fine Mapping of QTLs/QTNs and Mining of Genes Associated with Race 7 of the Soybean *Cercospora sojina* by Combining Linkages and GWAS

**DOI:** 10.3390/plants14131988

**Published:** 2025-06-29

**Authors:** Yanzuo Liu, Bo Hu, Aitong Yu, Yuxi Liu, Pengfei Xu, Yang Wang, Junjie Ding, Shuzhen Zhang, Wen-Xia Li, Hailong Ning

**Affiliations:** 1 Key Laboratory of Soybean Biology, Ministry of Education, Key Laboratory of Soybean Biology and Breeding/Genetics, Ministry of Agriculture, Northeast Agricultural University, Harbin 150030, China; 15645405369@163.com (Y.L.); hb554512341@163.com (B.H.); mdjyat@163.com (A.Y.); liuyuxi911@163.com (Y.L.); xupengfei@neau.edu.cn (P.X.); 2 College of Morden Agriculture and Ecological Environment, Heilongjiang University, Harbin 150080, China; 2004084@hlju.edu.cn; 3 Jiamusi Branch, Heilongjiang Academy of Agricultural Sciences, Jiamusi 154007, China; me999@126.com

**Keywords:** soybean, cultivar leaf spot, QTL, QTN, resistance gene

## Abstract

Soybean frogeye leaf spot (FLS) disease has been reported globally and is caused by the fungus *Cercospora sojina*, which affects the growth, seed yield, and quality of soybean. Among the 15 physiological microspecies of *C. sojina* soybean in China, Race 7 is one of the main pathogenic microspecies. A few genes are involved in resistance to FLS, and they cannot meet the need to design molecular breeding methods for disease resistance. In this study, a soybean recombinant inbred line (RIL3613) population and a germplasm resource (GP) population were planted at two sites, Acheng (AC) and Xiangyang (XY). Phenotypic data on the percentage of leaf area diseased (PLAD) in soybean leaves were obtained via image recognition technology after the inoculation of seven physiological species and full onset at the R3 stage. Quantitative trait loci (QTLs) and quantitative trait nucleotides (QTNs) were mapped via linkage analysis and genome-wide association studies (GWASs), respectively. The resistance genes of FLS were subsequently predicted in the linkage disequilibrium region of the collocated QTN. We identified 114 QTLs and 18 QTNs in the RIL3613 and GP populations, respectively. A total of 14 QTN loci were colocalized in the two populations, six of which presented high phenotypic contributions. Through haplotype–phenotype association analysis and expression quantification, three genes (Glyma.06G300100, Glyma.06G300600, and Glyma.13G172300) located near molecular markers AX-90524088 and AX-90437152 (QTNs) are associated with FLS Chinese Race 7, identifying them as potential candidate resistance genes. These results provide a theoretical basis for the genetic mining of soybean antigray spot No. 7 physiological species. These findings also provide a theoretical basis for understanding the genetic mechanism underlying FLS resistance in soybeans.

## 1. Introduction

Soybean frogeye leaf spot (FLS) is a disease reported globally and is caused by *Cercospora sojina*, which leads to the loss of seed yield. Owing to its complex pathogenesis, effectively controlling this disease is difficult. Therefore, resistance loci and genes need to be identified for the breeding of resistant varieties.

Physiological microspecies of soybean FLS differ, and genetic variation exists extensively; thus, classifying physiological microspecies is extremely important [1]. Experts and researchers worldwide have conducted physiological microspecies classification of soybean FLS [2,3,4,5]. In total, 20, 11, and 15 physiological microspecies of FLS have been identified in Brazil, the USA [6], and China [7], respectively.

Because of the diversity and variability of the physiological subspecies and the differences in pathogenesis and pathogenicity among different microspecies, the selected disease-resistant varieties may lose disease resistance because of the variation in physiological subspecies. To breed new disease resistance varieties, experts have identified the resistance genes of some physiological microspecies of FLS. Athow et al. bred the soybean variety “Lincoln” with the Rcs1 gene and its expression vector, which was resistant to the physiological microspecies American Race 1 [2]. The soybean variety “Kent” was bred, and the Rcs2 gene, which is resistant to the physiological microspecies American Race 2, was discovered [8]. Boerma et al. cultivated the soybean variety “Davis”, an expression vector of the American Rcs3 gene, which is resistant to the physiological microspecies Race 3 and various physiological subspecies known in Brazil and the United States [9]. In China, 15 Chinese races of FLS have been identified, among which Chinese Races 1, 7, and 10 are the dominant physiological races in northeast soybean production areas.

With advancements in molecular biology, breeders have mapped resistance-related loci and identified relevant genes that impart resistance to soybean FLS. Yang et al. obtained two markers closely associated with the resistance genes of FLS from the American variety “Peking” [10]. Gu et al. identified four quantitative trait nucleotides (QTNs) and four candidate genes controlling resistance to FLS Chinese Race 15 by conducting a genome-wide association study (GWAS) of 234 Chinese soybean varieties [11]. Di et al. evaluated resistance to Race 1 of soybean FLS via SSR marker-based association analysis with 202 representative soybean materials and identified 11 SSR loci significantly associated with resistance to Chinese Race 10 of FLS [12]. Na et al. assessed resistance to Race 7 of FLS in 335 representative soybean materials, identified 15 QTNs by conducting a GWAS, and screened four candidate genes regulating resistance to Chinese Race 7 of soybean FLS [13].

In most of the above studies, the FLS susceptibility level method was used to evaluate the resistance of soybean varieties to FLS at the disease level [10,11,12,13], and the results of disease level classification were affected by human factors with large errors. The percentage of leaf area diseased (PLAD) method, which quantifies the area of susceptible leaves via image recognition technology with less error than manual operation [14], was proposed to evaluate the resistance of the FLS.

Although the abovementioned studies have made considerable progress in gene mapping for resistance to different physiological races of soybean gray spot disease and have successfully identified multiple major genes, QTNs, and tightly linked markers, the resistance genes that have been mapped are mostly targeted at specific physiological races (such as Rcs1 against Race 1 and Rcs2 against Race 2). However, owing to the high diversity and variability of gray spot disease physiological races, the resistance mechanisms underlying existing resistance resources to emerging or composite races are unclear. Most studies have focused on single genetic populations or specific varieties (such as the American varieties ‘Lincoln’ and ‘Peking’ and local Chinese varieties), and the universality and environmental adaptability of resistance genes have not been fully analyzed. Additionally, there are still deficiencies in assessing complex genetic effects, such as polygenic interactions and QTN × environment interactions. Therefore, precise gene mapping is needed for dominant physiological races in different regions (such as Race 7).

This study used a soybean recombinant inbred line population, RIL3613, and a germplasm resource population, GP, to evaluate the resistance of Chinese Race 7 to PLAD. On the basis of the genotyping results obtained via the SNP660K (Beijing Boao Biotechnology Co., Ltd., Beijing, China). and Axiom_SoyaSNP chips (Beidahuang Kenfeng Seed Industry Co., Ltd., Harbin, China), linkage analysis and genome-wide association analysis of FLS resistance were performed. Potential candidate genes were mined in the colocalized genomic regions of the two populations, and then, haplotype and expression pattern analyses of different genotypes were performed via the resequencing data of the GP population. This study provides important information for understanding the genetic mechanism underlying soybean FLS resistance and for molecular-assisted breeding.

## 2. Materials and Methods

### 2.1. Genetic Material

A recombinant inbred line population, RIL3613, was used as the genetic material for quantitative trait locus (QTL) analysis of FLS. The population was crossed between the soybean varieties Dongnong L13 (DN L13, susceptible) and Heihe 36 (Hh 36, resistant) to obtain F_1_. For five consecutive generations after hybridization, the single-seed design method (SSD) was used for each generation. Finally, a recombinant inbred line population containing 120 homozygous genotype individuals was obtained [15]. The second population was a germplasm resource population (GP) containing four region-specific landraces endemic to Northeast China, 387 Chinese-bred cultivars, and 44 introduced cultivars of non-Chinese origin (see Supplementary Table S1 in Li et al. [15] for detailed accession profiles). This population was used as the genetic material for a GWAS on FLS.

### 2.2. Field Experiment

In 2022, the RIL3613 population and GP population were planted at the Xiangyang Experimental Practice Base (XY; 126.63° E, 45.75° N) and the Acheng Experimental Practice Base (AC; 127.63° E, 45.82° N) at Northeast Agricultural University. After inoculation, the average value (AVE) of the relative lesion area of the two populations in the two regions was measured. The planting density in both regions was 2.2 × 10^5^ plants/hm^2^, and the applied fertilizer concentrations of N/P_2_O_5_/K_2_O were 18/46/30 kg/hm^2^. A randomized block design with three replicates was used to conduct the field experiment. Each plot had a single ridge, with a ridge length of 3 m and a ridge spacing of 0.7 m. Single-row planting was conducted on ridges with a plant spacing of 0.07 m. The field management practices used were similar to local field management practices.

### 2.3. Inoculation of FLS

When the soybeans grew to the R3 stage, Chinese Race 7 FLS was obtained and preserved in the early stage of the study. The inoculated Chinese Race 7 strain was cultured on PDA medium (Potato Dextrose Agar, Hope Biotechnology Co., Ltd., Qingdao, China) at 25 °C for 7–10 days to obtain good activity. The colonies were isolated with a scalpel because more experimental materials needed to be inoculated. Chinese Race 7 was inoculated in half-cooked sorghum grains and cultured at 25 °C for one month to obtain more colonies. After maturation, the sorghum grains were gently washed with gauze in distilled water to separate the hyphae from the sorghum grains. The spore suspension obtained after scrubbing was passed through two layers of sterilized gauze to remove large hyphae and sorghum grains. The density of spores in the suspension was determined using a hemocytometer, and the concentration of spores was adjusted to 1 × 10^7^/mL. The concentration of sucrose in the spore suspension was 3%. After inoculation, the soybean plants were covered with a shade cloth. The humidity in the shade cloth was maintained at more than 90%, and the temperature was maintained below 30 °C. After 24 h, the inoculation process was repeated following the same steps, and the shade cloth was removed 24 h after the second inoculation.

### 2.4. Determination of the Diseased Leaf Area Ratio

At 20–30 days after the second inoculation, the FLS susceptibility status of each soybean plant was recorded. The four leaves showing the highest relative FLS infection area per variety were selected as four biological replicates. These leaves were photographed and numbered using a Canon DS126291 camera (Canon Inc., Taiwan, China). After the diseased spot pixels and the whole leaf pixels were obtained via image recognition technology via software labels (https://labelme.io/), the proportion of diseased spot pixels in the leaf pixels was calculated using the formula “PLAD = diseased spot pixels/leaf pixels”. Each leaf represented one independent replicate, resulting in four replicate measurements per variety. The average PLAD value per variety was used for subsequent analysis.

### 2.5. Phenotypic Variation Analysis

The average, coefficient of variation, kurtosis, skewness, normal distribution test, and analysis of variance of the RIL3613 population and GP population were calculated on the basis of the PLAD of the four leaves infected with FLS at the AC and XY sites.

Analysis of variance (ANOVA) was performed using data from four replicates in each environment. The ANOVA model included environment, genotype, and the environment ×genotype interaction effect. Genotype and environment × genotype interaction effects were considered random effects to estimate genotype, environment × genotype interaction variance, and error variance. The broad-sense heritability was subsequently calculated using the following formula:

$$h^{2}=\frac{\sigma_{G}^{2}}{\sigma_{G}^{2}+\frac{\sigma_{GE}^{2}}{e}+\frac{\sigma_{\varepsilon }^{2}}{re}}$$

where $h^{2}$ represents generalized heritability, $\sigma_{G}^{2}$ represents genotype variance, $\sigma_{GE}^{2}$ represents genotype-environment interaction variance, $\sigma_{ɛ}^{2}$ represents error variance, *e* represents the number of environments, and *r* represents the number of replicates.

The above analysis was performed via SAS 9.2 software (SAS Institute, Cary, NC, USA).

### 2.6. QTL Mapping

Young leaves of all lines from the RIL3613 population and its parents were frozen in liquid nitrogen and ground into a powder. Genomic DNA was extracted via the CTAB method. The DNA of the RIL3613 population material was used for SNP genotyping via a soybean SNP660K chip (Beijing Boao Biotechnology Co., Ltd., Beijing, China). SNPs with AA or BB frequency = 0, minor gene frequency < 0.05, sample loss rate > 0.1, or no clear clusters were removed, and the SNP markers obtained were used to make high-density genetic maps. The SNP markers with relatively good polymorphisms were used to construct the frame diagram, and an SNP marker was selected every 100 kb on the basis of the physical location of the SNP to construct the RIL3613 linkage diagram. After using Python 2.7 snpbinner (https://github.com/solgenomics/SNPbinner (accessed on 1 February 2021)), RIL3613 group SNP data were screened to identify possible intersections (minimum distance between the intersection points 0.2% of the chromosome length). The aggregation breakpoints generated by the intersections were subsequently used to create representative bins for the entire population, with a minimum distance of 30 kb per bin, resulting in 2177 bins. The SNPbin high-density genetic linkage map was constructed via IciMapping 4.1 software (https://isbreeding.caas.cn/rj/qtllcmapping/ (accessed on 1 February 2021)), and the entire SNP was anchored on 20 linkage groups covering 3539.66 cM. Each chromosome had 1031 segments, ranging from 13 to 79. The interval of each chromosome ranged from 1.92 cM to 10.93 cM, with a total mean of 4.09 cM [16]. On the basis of the ratio of the physical distance (bp) between the anchored first and last markers to the entire chromosome length (bp), the map covered 40.12% to 99.81% of each chromosome. Among all the intervals between the markers, 87 were more than 10 cM apart, accounting for 39.26% of the total length of the genetic map.

On the basis of the bin genetic map obtained, the phenotypic data and the average PLAD of FLS at two sites of the RIL3613 population were used to map the QTLs. IciMapping 4.0 software was used for QTL mapping via inclusive composite interval mapping (ICIM) [17]. The LOD threshold was set to 2.5, and the PIN value was set to 0.01.

### 2.7. Genome-Wide Association Analysis

Young leaves of all varieties in the GP population were frozen in liquid nitrogen and ground into a powder. Genomic DNA was extracted using the CTAB method. DNA from the GP population samples was used for SNP typing using an Axiom_SoyaSNP chip (Beidahuang Kenfeng Seed Industry Co., Ltd., Harbin, China). Based on the criteria of a minor allele frequency > 0.05 and a maximum missing frequency of SNPs < 10%, SNP genotyping was performed, and 63,306 high-quality SNPs were obtained [18].

Using TASSEL5.0 software, the linkage disequilibrium value (r^2^) of all pairs of SNP markers within a physical distance of 10 Mb for 20 chromosomes was calculated. At the physical position where the negative natural logarithm of r^2^ dropped to half of the maximum value, the LD attenuation distance was estimated to be 86 kb [16]. To avoid the effects of kinship and population structure on the results of genome-wide association analysis, kinship and population structure analyses were performed, and the GP population was divided into two subgroups.

The PLAD data of FLS in the GP populations obtained from the two environments were used as phenotypic data. First, the 3VmrMLM was used to conduct genome-wide association analysis on the PLAD of FLS Race 7 in single environments of GP populations. The phenotypic data of the two environments were analyzed together to obtain the general QTN and QEI of the two environments. The analysis used the R language software package 3VmrMLM [19,20].

### 2.8. Prediction of Candidate Genes

For the LD attenuation distance of the SNPs (43 kb genome in the SNP flank area) colocalized via linkage analysis and genome-wide association studies, all genes were retrieved from the Pytozome website (https://phytozome-next.jgi.doe.gov/ (accessed on 1 February 2021)). The hierarchy files of GO and background files of KEGG were downloaded via TBtools-II (Toolbox for Biologists v2.119) software. The number of proteins associated with the preliminary predicted candidate genes was searched in the NCBI database (https://www.ncbi.nlm.nih.gov/ (accessed on 1 February 2021)), and the total protein files of the soybeans were downloaded. The whole-genome protein functions were annotated via eggNOG-mapper (http://eggnog-mapper.embl.de/ (accessed on 1 February 2021)).

### 2.9. Analysis of Haplotypes

Among the 455 soybean varieties in the GP population, 203 were selected for whole-genome resequencing. The allelic genotypes of the candidate genes were classified on the basis of comparisons between the sequences of the candidate genes of the 203 soybean varieties and those of Williams82 in the NCBI (https://www.ncbi.nlm.nih.gov/ (accessed on 1 February 2021)) database. Combining the phenotypic data of the PLAD of the 203 varieties in the GP population after inoculation with FLS Race 7, the difference in the significance of the PLAD between different alleles was tested via GraphPad Prism 9.0 software (https://www.graphpad-prism.cn/ (accessed on 1 February 2021)).

### 2.10. Analysis of Candidate Gene Expression

We performed quantitative real-time polymerase chain reaction (qRT-PCR) to analyze the resistant variety “Heinong47” (resistant to Chinese Race 7, carrying all the beneficial alleles of FLS resistance SNPs) and the susceptible variety “Dongnong L13” (susceptible to Chinese Race 7, carrying none of the beneficial alleles of FLS resistance SNPs). At 0, 1, 3, and 6 h after inoculation, the leaves of the soybean plants were sampled, and three biological replicates were taken. The FastPure Universal Plant Total RNA Isolation Kit (Nanjing Novozam Biosciences Co., Ltd., Nanjing, China) was subsequently used to extract RNA from the samples, and the HiScript^®^ III 1st Strand cDNA Synthesis Kit (+gDNA wiper) (Nanjing Novozam Biosciences Co., Ltd., Nanjing, China) was subsequently used for reverse transcription to synthesize cDNA. The qRT-PCR assay was performed using a Roche LightCycler^®^ 480 real-time fluorescence quantitative PCR apparatus (F. Hoffmann-La Roche, Ltd., Rotkreuz, Switzerland) and a SuperReal PreMix Plus (SYBR Green) kit (Nanjing Novozam Biosciences Co., Ltd., Nanjing, China). The experiment was repeated three times. The sequences of the primers used to amplify the candidate genes are listed in Table S1.

### 2.11. Analysis of Haplotype Distribution

Based on the resequencing data of 2883 germplasm resources (including 1733 cultivated soybean varieties, 1048 local soybean varieties, and 102 wild soybean varieties) from the National Biological Information Center database (https://ngdc.cncb.ac.cn/soyomics/index (accessed on 6 March 2025)), the proportions of target gene haplotypes in cultivated, landrace, and wild soybean cultivars were determined.

### 2.12. Methods for Predicting Protein Tertiary Structures Corresponding to Different Haplotypes of Candidate Genes

The amino acid sequences encoded by genes were downloaded from the Phytozome website (https://phytozome-next.jgi.doe.gov/ (accessed on 2 April 2025)), the mutated amino acid sequences were calculated via another website (https://www.novopro.cn/tools/translate.html (accessed on 2 April 2025)), and tertiary structure prediction of different proteins encoded by different genotypes of the same gene was performed using the alphafold3 website (https://alphafoldserver.com/ (accessed on 2 April 2025)). The prediction results were visualized and analyzed using PyMOL(TM) 3.1.5.1 software.

### 2.13. Promoter Cis-Acting Element Prediction

The 2000 bp promoter sequence upstream of the gene CDS was downloaded from the Phytozome website (https://phytozome-next.jgi.doe.gov/ (accessed on 2 April 2025)), the cis-acting elements in the promoter were predicted using the PlantCARE website (https://bioinformatics.psb.ugent.be/webtools/plantcare/html/ (accessed on 2 April 2025)), and the results of the analysis were visualized using Tbttools - II (Toolbox for Biologists) v2.309 software.

## 3. Results and Analysis

### 3.1. Phenotypic Variation in the PLAD of FLS Race 7 in the Two Populations

The RIL3613 and GP populations were planted in two environments, and phenotypic data were recorded and subjected to descriptive analysis after inoculation at the R3 stage.

For the RIL3613 population, the relative lesion areas of the parent lines DN L13 and Heihe 36 were 0.015 and 0.009, respectively, in the XY environment and 0.038 and 0, respectively, in the AC environment. These findings indicate that the incidence of gray spot disease is influenced by the environment. The average relative lesion area of gray spot disease in RIL3613 was smaller in the XY environment than in the AC environment, suggesting that the development of FLS was more prominent in the AC environment. The phenotypic skewness and kurtosis of the relative lesion area were 5.238 and 35.731 in the XY environment and 3.371 and 15.099 in the AC environment, respectively, indicating that the relative lesion area data of FLS showed a right-skewed distribution. Most lines presented lesion areas concentrated in the lower range, with only a few lines exhibiting high susceptibility (Table 1).

For the GP population, soybeans’ average relative lesion areas were 0.031 and 0.027 in the XY and AC environments, respectively, indicating a more apparent incidence of disease in the XY environment. The minimum value of the relative lesion area was 0 in the XY and AC environments, suggesting adequate resistance in some lines. The maximum relative lesion areas were 0.103 and 0.163 in the AC and XY environments, respectively, indicating poor resistance and severe disease incidence in some lines. The phenotypic skewness and kurtosis of the relative lesion area were 1.224 and 1.398 in the XY environment and 1.092 and 0.404 in the AC environment, respectively, suggesting that the relative lesion area data of gray spot disease showed a right-skewed distribution but with a smaller degree of deviation and a more moderate data distribution than those of the RIL3613 population (Table 2).

The results of the ANOVA on PLAD in the two environments of XY and AC for the RIL3613 and GP populations revealed that the differences in the environmental and genotype variances and the genotype–environment interaction effects were extremely significant (Tables S2 and S3). The extremely significant genotypic variance indicated that there was high genetic variation in the two populations. The highly significant interaction variance between the environment and genotype indicated that the expression of FLS resistance genes differed in different environments. The heritability of the RIL3613 population was greater in a single environment and lower in multiple environments, indicating that the environment had a greater effect on gene expression. The single-environment heritability of the GP population was greater than that of the single-environment population. In contrast, the multiple-environment heritability was greater than that of the single-environment population. These findings indicate that environmental factors have a limited effect on gene expression.

### 3.2. QTLs for FLS in the RIL3613 Population

QTL mapping was performed in the RIL3613 population via ICIM based on the genetic map. We identified 90 QTLs associated with the resistance of FLS Chinese Race 7 in the two environments (Table S4).

**Figure 1:**
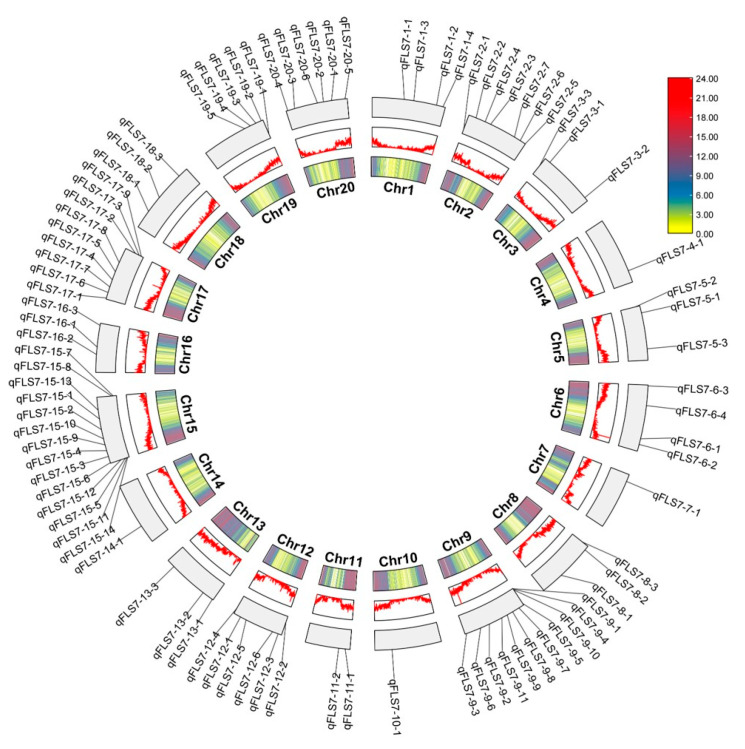
Distribution of PLAD QTLs on 20 chromosomes during infection of the soybean FLS Race 7. Note: The innermost circular plot depicts the soybean gene density heatmap (the color gradient represents the number of genes per megabase (genes/Mb)), the middle ring illustrates the gene density frequency distribution, and the outermost ring visually maps the localization of 90 PLAD-associated QTLs across 20 chromosomes in this study. The chromosome nomenclature is exemplified by qFLS7-1–3: the prefix “qFLS7” denotes a quantitative trait locus (QTL) for resistance to FLS Chinese Race 7, the middle digit “1” indicates the chromosome number, and the suffix “3” designates the QTL identifier on that chromosome.

**Figure 2:**
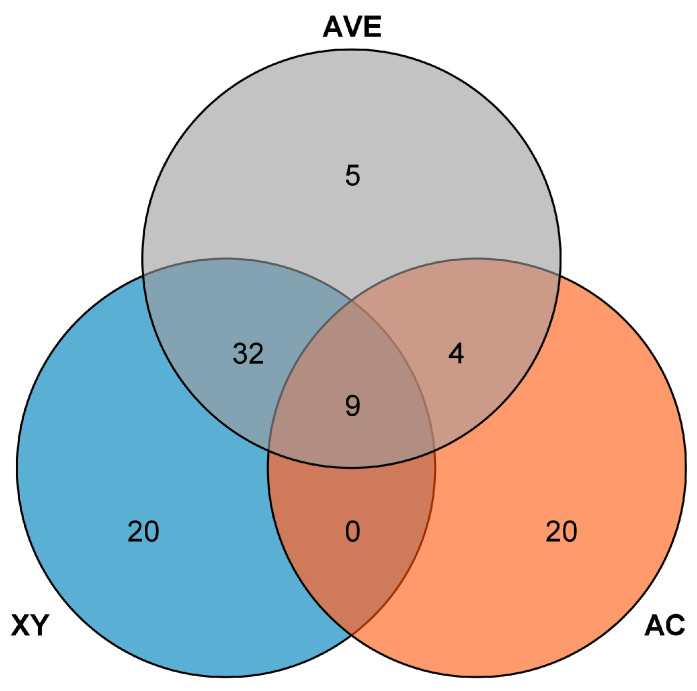
Venn diagram of the number of PLAD QTLs mapped among the two environments and the average number of PLAD QTLs mapped. Note: AC: quantity of QTLs mapped based on the PLAD phenotypic data in the single environment of AC; XY: The quantity of QTLs mapped based on the PLAD phenotypic data in the single environment of XY. AVE: The quantity of QTLs mapped based on the mean value of PLAD phenotypic data from the AC and XY environments. The overlapping region denotes the number of QTLs identified through repeated mapping (i.e., colocalized QTLs across different environments).

The QTLs were distributed on soybean chromosomes 1 (4), 2 (7), 3 (3), 4 (1), 5 (3), 6 (4), 7 (1), 8 (3), 9 (11), 10 (1), 11 (2), 12 (6), 13 (3), 14 (1), 15 (14), 16 (3), 17 (9), 18 (3), 19 (5), and 20 (6) (Figure 1). Among them, 33 QTLs were identified in the AC environment and were distributed on 16 chromosomes, with LOD scores ranging from 2.6 to 21.9, and a single QTL explained 1.3–5.3% of the phenotypic variation. We identified 61 QTLs in the XY environment, which were distributed on 18 chromosomes, with LOD scores ranging from 2.7 to 16.1, and a single QTL explained 0.1–0.38% of the phenotypic variation. In total, 50 QTLs were mapped through the average of two environments, distributed on 15 chromosomes, with LOD values ranging from 2.8 to 12, and a single QTL explained 0.37–0.9% of the phenotypic variation. Additionally, 36 QTLs were co-detected in two environments and 9 QTLs were co-detected in three environments. (Figure 2, Table 3, Table S4).

### 3.3. QTN for the PLAD Identified in the GP Population

**Figure 3:**
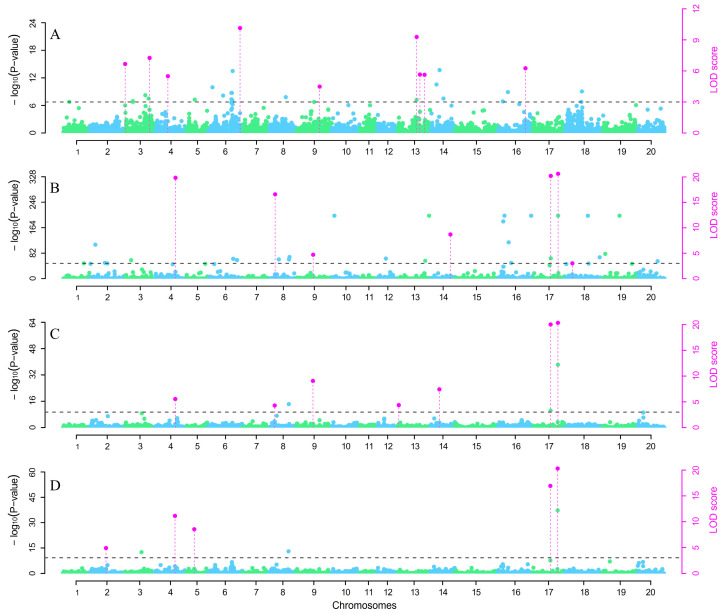
Manhattan plot of the genome-wide association study of PLAD in the GP populations. (**A**): QTN detected in the AC environment; (**B**): QTN detected in the XY environment; (**C**): Main QTN in the two environments; (**D**): QTN by environment interactions (QEIs) in the two environments. Note: Green and blue dots represent the distribution of single nucleotide polymorphisms (SNPs) across the chromosomes; Pink dots indicate SNPs with high −log10(*p*-value) and LOD score values at specific chromosomal positions; Pink vertical lines indicate the precise locations of significantly associated SNPs on the chromosomes.

The 3VmrMLM GWAS method was used to analyze 455 GP populations in a single environment and a combination of the two environments. A total of 18 QTNs significantly associated with FLS were detected on chromosomes 2 (1), 3 (2), 4 (2), 5 (1), 6 (1), 8 (1), 9 (2), 13 (3), 14 (1), 16 (1), 17 (2), and 18 (1). The seven QEI significantly associated with FLS were distributed on seven chromosomes, including chromosomes 4 (1), 8 (1), 9 (1), 13 (1), 14 (1), and 17 (2) (Figure 3, Table 4). Among them, nine QTNs were located in the XY environment, and a single QTN explained 2.21–6.94% of the phenotypic variation, accounting for 45.01% of the total. Seven QTNs were identified in the environment AC, and a single QTN explained 0.94–49.19% of the phenotypic variation, accounting for 78.53% of the total. Eight QTNs and seven QEIs were mapped in the joint analysis of the two environments, accounting for 62.07% of the phenotypic variation; a single QTN explained 1.3–17% of the phenotypic variation, whereas a single QEI explained 1.1–18.4% of the phenotypic variation (Table 4). Among them, Ax-90468600 (chromosome 4), Ax-90506929 (chromosome 9), AX-90517572 (chromosome 17), and AX-90437152 (chromosome 17) were located in the environment AC and average of the two environments. AX-90517572 and AX-90437152 presented relatively high phenotypic contribution rates.

### 3.4. Candidate Genes for Resistance in FLS Race 7

**Figure 4:**
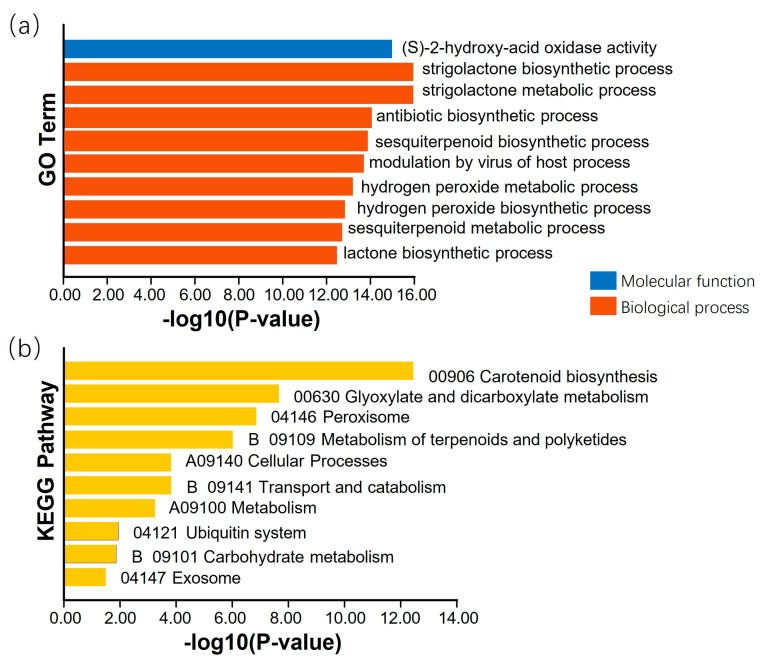
KEGG and GO analyses. Note: The X-axis represents -log_10_ (*p* value), reflecting the enrichment significance of the GO/KEGG terms. Larger values indicate smaller *p* values and more significant enrichment. (**a**) GO enrichment. The Y-axis presents GO terms categorized into molecular functions (blue bars) and biological processes (red bars), illustrating significantly enriched biological functional categories. (**b**) KEGG enrichment. The Y-axis lists KEGG pathway names, sorted by categories such as metabolic pathways and signal transduction, to display significantly enriched biological pathways.

Combining the results of the genome-wide association analysis and linkage analysis, 14 QTNs or QEIs detected in the GP were colocalized within the 11 QTL intervals of RIL3613, including AX-90325055, AX-90319684, AX-90524088, AX-90332112, AX-90339041, AX-90443776, AX-90506929, AX-90430118, AX-90524893, and AX-904396, AX-90463766, AX-90517572, AX-90437152, and AX-90433524 (Table 5 and Figure S1). Within the LD decay distance, 85 genes were found. Through the combination of KEGG and GO analyses (Figure 4) and functional annotation information, 15 genes were predicted to be related to the resistance of FLS Race 7 (Table 6).

To further screen candidate genes, the sequences of the above genes of the 203 varieties in the GP were compared with those of the Williams82 variety in the NCBI database. The results revealed that five genes simultaneously had non-synonymous mutations in exons and the promoter region (Table 7 and Table S5), whereas 11 genes had mutations only in the promoter region (Table S5). Therefore, these 11 genes could be preliminarily predicted as candidate genes regulating the resistance of FLS Race 7.

### 3.5. Validation of Candidate Genes

Combined with the PLAD obtained from the inoculation of 203 germplasm resources with physiological Race 7 of soybean gray spot disease, haplotype analysis was performed on the candidate genes.

**Figure 5:**
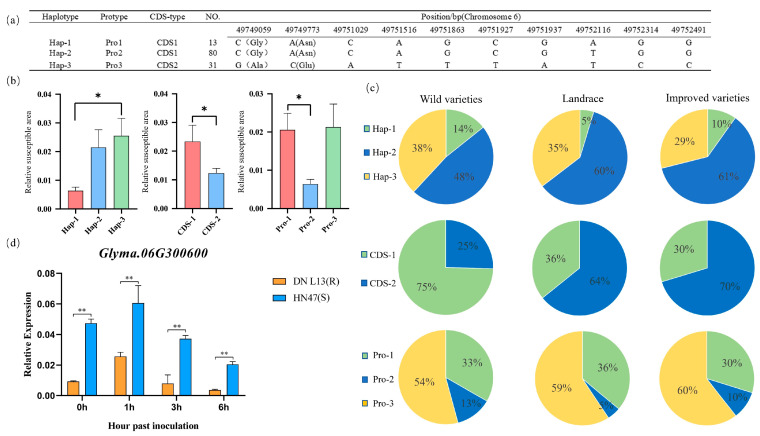
Analysis of the haplotype and expression level of the *Glyma.06G300600* gene. (**a**) Information on the mutation of the *Glyma.06G300600* gene. (**b**) Haplotype analysis of *Glyma.06G300600*. Note: The bar height denotes the relative susceptible area. Different colors distinguish sample groups: Hap-1/Hap-2/Hap-3 represent distinct gene haplotypes; CDS-1/CDS-2 are coding sequences; and Pro-1/Pro-2/Pro-3 denote promoter regions. A t-test (and non-parametric tests) was used to assess intergroup differences, with * indicating *p* < 0.05. (**c**) Proportion of different haplotypes of the *Glyma.06G300600* gene in three types of varieties. (**d**) Analysis of the expression pattern of the *Glyma.06G300600* gene. Note: Bar height denotes the mRNA level of each candidate gene in the resistant soybean variety (HN 47) and the susceptible soybean variety (DN L13). These values are the averages of three biological replicates. A t test (and non-parametric tests) was used to assess intergroup differences, with ** indicating *p* < 0.01.

The *Glyma.06G300600* gene has two SNP mutations in the exon region, leading to the substitution of glutamic acid at position 279 with asparagine and the substitution of alanine at position 504 with glycine in the encoded protein. This allows the gene to be divided into two haplotypes, namely CDS-1 and CDS-2. In the promoter region, eight base mutations result in three haplotypes, designated Pro-1, Pro-2, and Pro-3. By combining the variations in the exons and promoters of the *Glyma.06G300600* gene, three gene haplotypes were identified: Hap-1, Hap-2, and Hap-3 (Figure 5a). The PLAD after inoculation was significantly greater in the CDS-1 type than in the CDS-2 type; the PLAD of the Pro-1 type was significantly greater than that of the Pro-2 type; the PLAD of the Hap-3 type was significantly greater than that of the Hap-1 type, whereas the PLAD of Hap-2 was between those of Hap-1 and Hap-3. Therefore, the CDS-2, Pro-2, and Hap-1 types presented stronger resistance to Physiological Race 7 of soybean gray spot disease and were excellent haplotypes for soybean resistance to Physiological Race 7 of gray spot disease (Figure 5b).

By analyzing resequencing data of 2883 accessions from the National Center for Biotechnology Information (https://ngdc.cncb.ac.cn/soyomics/index (accessed on 6 March 2025)) database, we investigated the distribution patterns of different haplotypes in three soybean varieties: wild soybeans, landraces, and improved varieties. The percentages of the superior haplotypes of the *Glyma.06G300600* gene (CDS-2, Pro-2, and Hap-1) were 25%, 13%, and 14%, respectively, in wild soybeans; 64%, 5%, and 10%, respectively, in landraces; and 70%, 10%, and 14%, respectively, in improved varieties. The CDS-2 haplotype was significantly enriched in landraces and improved varieties, probably because breeders preferentially retain varieties carrying this haplotype because of its pronounced disease resistance. In contrast, the Pro-2 and Hap-1 frequencies remained relatively stable across all three groups without significant changes (Figure 5c).

Owing to mutations in the promoter of the *Glyma.06G300600* gene, to investigate the role of promoter variation, the expression levels of this gene were detected in the disease-susceptible variety (DN L13) and disease-resistant variety (HN 47) after inoculation. The results of the qRT-PCR analysis of the changes in the expression of the *Glyma.06G300600* gene revealed that gene expression first increased but then decreased, peaking at 1 h after inoculation. The expression level in HN47 was significantly greater than that in DN L13. We predicted that this gene has a positive regulatory effect on disease resistance (Figure 5d).

**Figure 6:**
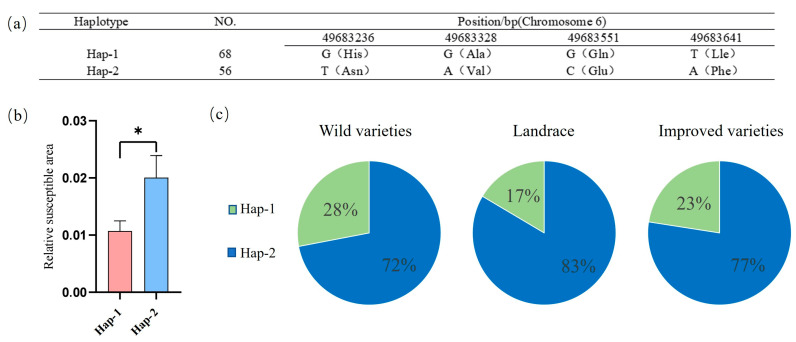
Haplotype analysis of the *Glyma.06G300100* gene. (**a**) Information on the mutation of the *Glyma.06G300100* gene. (**b**) Haplotype analysis of the *Glyma.06G300100* gene; * represents *p* < 0.05. Haplotype analysis of *Glyma.06G300100*. Note: The bar height denotes the relative susceptible area. Different colors distinguish sample groups, and Hap-1 and Hap-2 represent distinct haplotypes of the gene. A t test (and non-parametric tests) was used to assess intergroup differences, with * indicating *p* < 0.05. (**c**) Proportion of different haplotypes of the *Glyma.06G300100* gene in three types of variety.

The *Glyma.06G300100* gene has four SNP mutations in the exon region, leading to the substitution of isoleucine at position 152 with phenylalanine, glutamine at position 182 with glutamic acid, alanine at position 256 with valine, and histidine at position 287 with aspartic acid. This allows the gene to be divided into two haplotypes, designated Hap-1 and Hap-2 (Figure 6a). The PLAD after inoculation was significantly greater in the Hap-2 type than in the Hap-1 type. Therefore, the Hap-1 type is an excellent haplotype for soybean resistance to the physiological Race 7 of gray spot disease (Figure 6b).

An analysis was conducted on the proportions of the two haplotypes of the *Glyma.06G300100* gene in three categories of soybean varieties: wild soybeans, landraces, and improved varieties. The excellent haplotype Hap-1 accounted for 28%, 17%, and 23% of the wild soybean, landrace, and improved varieties, respectively. The lower proportion of Hap-1 in landraces may be attributed to the fact that landraces prioritize high-yielding soybeans while neglecting resistance to gray spot disease (Figure 6c).

**Figure 7:**
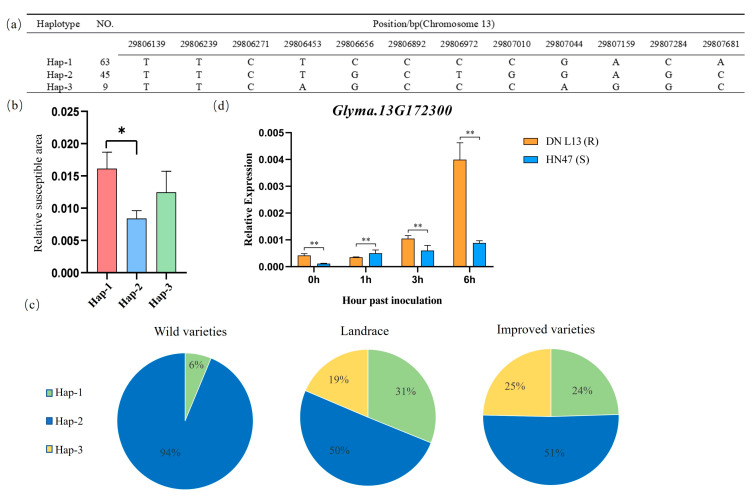
Analysis of the haplotype and expression level of the *Glyma.13G172300* gene. (**a**) Information on the mutation of the *Glyma.13G172300* gene; (**b**) haplotype analysis of the *Glyma.13G172300* gene. Note: The bar height denotes the relative susceptible area. Different colors distinguish sample groups, and Hap-1, Hap-2, and Hap-3 represent distinct gene haplotypes. A t-test (and non-parametric tests) was used to assess intergroup differences, with * indicating *p* < 0.05. (**c**) The proportion of different haplotypes of the *Glyma.13G172300* gene in three types of varieties. (**d**) Analysis of the expression pattern of the *Glyma.13G172300* gene. Note: Bar height denotes the mRNA level of each candidate gene in the resistant soybean variety (HN 47) and the susceptible soybean variety (DN L13). These values are the averages of three biological replicates. A t-test (and non-parametric tests) was used to assess intergroup differences, with ** indicating *p* < 0.01.

The *Glyma.13G172300* gene has 12 base mutations in the promoter region, which divides the gene into three haplotypes (Figure 7a): Hap-1, Hap-2, and Hap-3. Among them, the PLAD after inoculation in the Hap-1 type of *Glyma.13G172300* was significantly greater than that in the Hap-2 type, and the PLAD of the Hap-3 type was intermediate between those of Hap-1 and Hap-2. Therefore, the Hap-2 haplotype is highly resistant to physiological Race 7 of soybean gray spot disease and is an excellent haplotype for soybean resistance to physiological Race 7 of gray spot disease (Figure 7b).

An analysis was performed on the proportions of the three haplotypes of the *Glyma.13G172300* gene in three categories of soybean varieties: wild soybeans, landraces, and improved varieties. The excellent haplotype Hap-2 accounted for 94%, 50%, and 51% of the haplotypes in wild soybeans, landraces, and improved varieties, respectively (Figure 7c). It had the highest proportion in wild varieties and lower in landraces and improved varieties. The high frequency of Hap-2 in wild soybeans reflects the retention of its adaptive advantages through natural selection. At the same time, its lower proportions in landraces and improved varieties may be attributed to changes in the direction of artificial selection.

Owing to mutations in the promoter of the *Glyma.13G172300* gene, its expression level was detected. The results of the qRT-PCR assays revealed that the expression of the *Glyma.13G172300* gene increased within 6 h after soybean inoculation, peaking at 6 h post-inoculation. The expression level in the disease-susceptible variety DN L13 was significantly greater than that in the disease-resistant variety HN47. We hypothesized that this gene negatively regulates disease resistance (Figure 7d).

### 3.6. Protein Tertiary Structure Prediction

**Figure 8:**
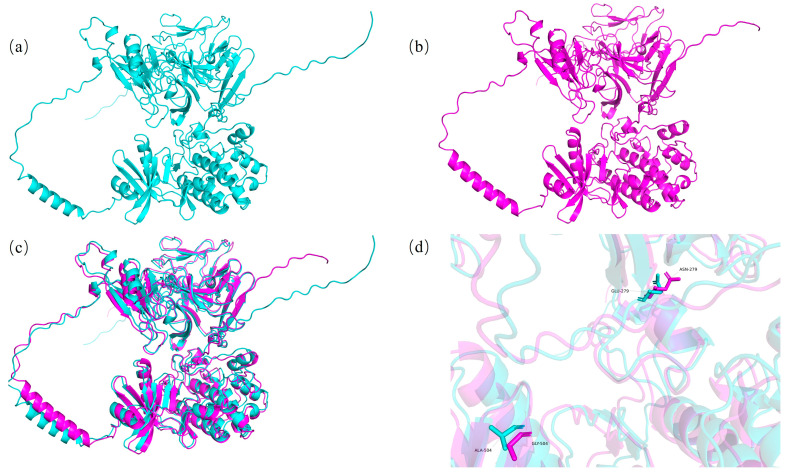
Comparison of the tertiary structures of proteins with different genotypes of the *Glyma.06G300600* gene. (**a**) Tertiary structure of the CDS-1-type protein. (**b**) Tertiary structure of the CDS-2-type protein. (**c**) Overlay diagram of the tertiary structures of the CDS-1 and CDS-2 types. (**d**) Binding sites affecting the tertiary structure of the protein.

To calculate the amino acid sequences of the CDS-1 and CDS-2 genotypes of the *Glyma.06G300600* gene and the Hap-1 and Hap-2 genotypes of the *Glyma.06G300100* gene, a website (https://www.novopro.cn/tools/translate.html (accessed on 2 April 2025)) was used. The results revealed that in the amino acid sequence encoded by the *Glyma.06G300600* gene, the alanine at position 504 was mutated to Glycine, and the glutamic acid at position 279 was mutated to asparagine. We hypothesized that this may affect the tertiary structure of the translated protein, thereby influencing the regulatory role of this gene in Physiological Race 7 of soybean gray spot disease. The Alphafold3 website (https://alphafoldserver.com/) was used to predict the tertiary structures of the proteins of the CDS-1 and CDS-2 genotypes. The model with the highest confidence was selected as the prediction model. The prediction results were visually analyzed via PyMol software, which revealed that the tertiary structures of the proteins of the CDS-1 and CDS-2 genotypes of the *Glyma.06G300600* gene were different (Figure 8).

**Figure 9:**
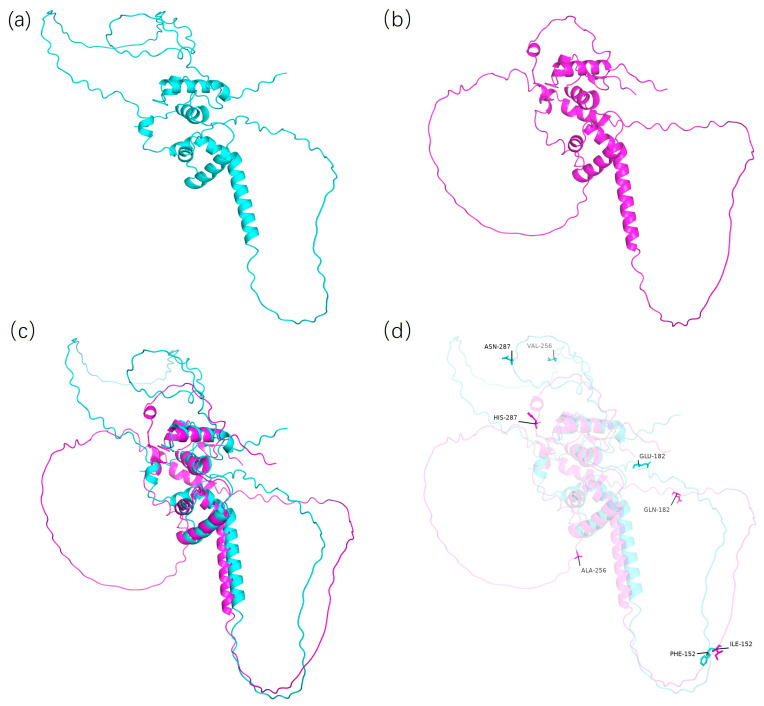
Comparison of the tertiary structures of proteins with different genotypes of the *Glyma.06G300100* gene. (**a**) Tertiary structure of the Hap-1-type protein. (**b**) Tertiary structure of the Hap-2-type protein. (**c**) Overlay diagram of the tertiary structures of the Hap-1 and Hap-2 types. (**d**) Binding sites affecting the tertiary structure of the protein.

The amino acid sequence encoded by the *Glyma.06G300100* gene was as follows: the isoleucine at position 152 was mutated to phenylalanine; the glutamine at position 182 was mutated to glutamic acid; the alanine at position 256 was mutated to valine; and the histidine at position 287 was mutated to aspartic acid. Using the above-described methods, the tertiary structures of the proteins from the Hap-1 and Hap-2 genotypes of the *Glyma.06G300100* gene were predicted and visually analyzed. The prediction results revealed prominent differences in the tertiary structures of the Hap-1 and Hap-2 genotype proteins (Figure 9).

### 3.7. Analysis of Promoter Cis-Acting Elements

**Figure 10:**
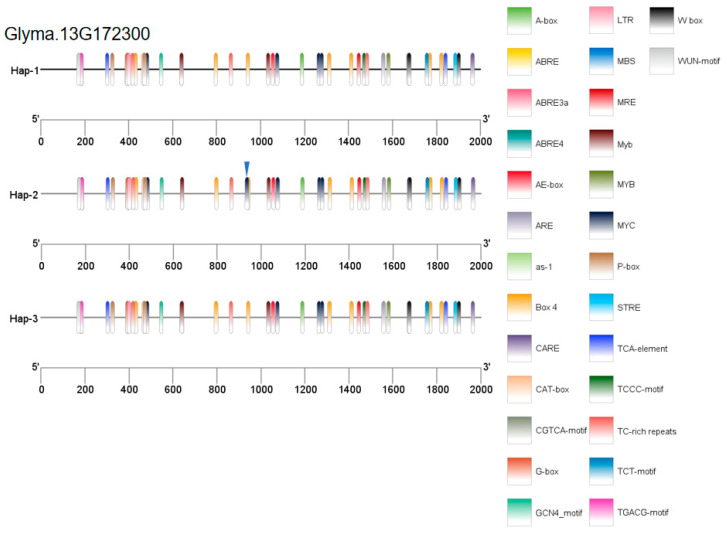
Analysis of the promoter elements of the *Glyma.13G172300* gene.

Owing to mutations in the promoter of the *Glyma.13G172300* gene, we hypothesized that these mutations may alter cis-acting elements and thus affect gene function. The PlantCARE website (https://bioinformatics.psb.ugent.be/webtools/plantcare/html/ (accessed on 2 April 2025)) was used to predict cis-acting elements in the promoters of the three genotypes of the *Glyma.13G172300* gene, and TBtools-II software was used for visual analysis. A C-to-G mutation at position 1446 bp upstream of the gene introduces an additional MYC cis-acting element in the promoter. This may increase the sensitivity of the gene to MYC transcription factors, such that low levels of MYC can trigger significant gene expression. One study reported that MYC transcription factors are involved in plant stress resistance and serve as key regulators of the jasmonic acid signaling pathway [21]. This finding agreed with the haplotype analysis results in this experiment, where the Hap-2 type exhibited significantly stronger resistance than the Hap-1 type (Figure 7b). Therefore, we hypothesized that changes in cis-acting elements at this locus may affect the expression level of *Glyma.13G172300* (Figure 7d), thereby affecting plant resistance to Physiological Race 7 of soybean gray spot disease (Figure 10).

**Figure 11:**
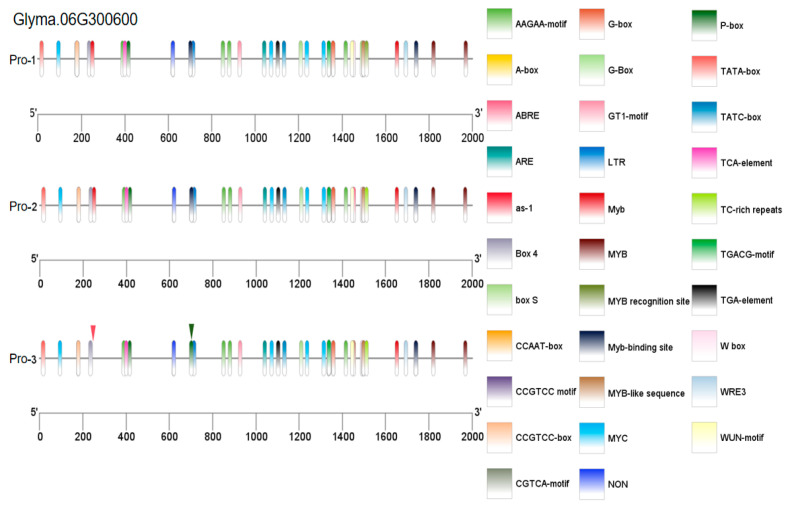
Analysis of the promoter elements of the *Glyma.06G300600* gene.

Owing to mutations in the promoter of the *Glyma.06G300600* gene, the above methods were used to predict and visually analyze the promoter elements of the gene. A C-to-A mutation at position 1296 bp upstream of the gene caused the MYB-binding site cis-acting element in the promoter to change to a P-box cis-acting element. A C-to-G mutation at position 1745 bp upstream led to the deletion of a MYB cis-acting element in the promoter, reducing the regulatory effect of MYB transcription factors on this gene. MYB family transcription factors play important roles in plant responses to biotic stress [22]. This finding is consistent with the haplotype analysis results in this study, in which the Pro-2 type was the dominant haplotype and exhibited stronger resistance than the Pro-3 type (Figure 5b). Therefore, we hypothesized that changes in cis-acting elements at these two positions may affect the expression level of *Glyma.06G300600* (Figure 5d), thereby influencing plant resistance to the physiological Race 7 of soybean gray spot disease (Figure 11).

## 4. Discussion

### 4.1. Genetic Characteristics of the PLAD of FLS in Soybean

The evaluation indicators for soybean FLS disease include the disease resistance index method [11,13,23], visual grading [10,14,24], lesion number [14], and PLAD [14]. Among these four methods, PLAD is achieved via image recognition to calculate the diseased area and total area of leaves. Since the phenotypic calculation part of FLS does not require human involvement, it reduces the influence of subjective human factors. In this study, the PLAD of the Chinese Race 7 FLS disease in RIL3613 and the GP population had a skewed continuous distribution in various environments, which indicated that multiple genes control the PLAD and that the expression of genes is strongly affected by the environment (Tables S2 and S3). This finding is similar to the results reported by McDonald et al. [14]. The mapping results revealed that 90 QTLs were mapped in the RIL3613 population, whereas 19 QTNs were located in the GP population. Similarly, McDonald et al. [14] located 11 QTNs. These findings indicate that PLAD is controlled by multiple genes.

### 4.2. Integration of Linkage Analysis and 3VmrMLM Enhances the Accuracy of Mapping Genes Associated with Soybean FLS PLAD

The results of previous studies and this study demonstrated that the PLAD of soybean FLS is a complex polygenic and environmentally sensitive trait. Consequently, genetic mapping is influenced by population structure, statistical methodologies, and experimental environments. Linkage analysis of recombinant inbred line (RIL) populations relies on interval mapping via genetic maps, which exhibit low detection accuracy as primary mapping methods [25]. In this study, QTL mapping for PLAD using the RIL3613 population identified 90 QTLs, with individual QTLs explaining 0.1–5.3% of the phenotypic variation. This may be because the interval contains multiple genes associated with PLAD, and the genetic factors influence each other, resulting in a low phenotypic contribution rate of a single interval. Additionally, the environment affects the expression of genes. Subsequently, it affects the genetic effects of QTLs in different environments, resulting in large differences in the results of QTL mapping between different environments. Only nine of the 90 QTLs detected in the RIL3613 population were consistently identified across the two environments, whereas the remaining 81 were environment specific. These environment-specific QTLs were detectable only in environments where their genetic effects were pronounced, reflecting a neglect of gene × environment (G × E) interaction effects in conventional linkage analysis. Similarly, GWAS using germplasm populations enables higher-resolution detection of genome-wide SNPs associated with target traits and more accurate estimation of minor genetic effects. Integrating GWAS with linkage analysis can refine the mapping precision of loci associated with infected PLAD in the RIL3613 population. This approach has been applied to fine-map QTLs and to identify candidate genes for soybean protein content [26], soybean mosaic virus disease [27], and salt-alkali tolerance [28]. The 3VmrMLM [20] was used in this study to address multi-environment QTN detection. This method enables simultaneous identification of the main effect and QTN × environment interaction (QEI) effects and is widely used for dissecting complex traits in crops [29,30,31,32,33,34,35]. The 3VmrMLM was used to analyze PLAD data in single and joint environments, revealing 16 environment-specific QTNs, five main QTNs, and seven QEIs. Notably, 14 PLAD-associated QTNs colocalized with 11 QTLs identified in the RIL3613 population, which finely mapped genomic regions to search for PLAD-related genes in the RIL3613 population.

### 4.3. Colocalization of QTL/QTN with Different Genetic Backgrounds

By comparing the 14 QTN/QEI loci located within 11 QTL intervals with the disease resistance loci identified in the SoyBase database, nine were found to be adjacent to the previously reported disease resistance loci. Among them, AX-90470366, located on chromosome 3, is close to ss715586200, which is resistant to Race 3 [14]. AX-90319684 on chromosome 5 is close to Afx-89062122 and Afx-89220750, which are resistant to FLS Chinese Race 15 [11]. AX-90524088 on chromosome 6 is adjacent to Gm06:4218717, which is resistant to Chinese Race 7 [14], and Gm06:42106014, which is resistant to Chinese Race 1 [23]. On chromosome 9, AX-90443776 is adjacent to Satt260, which is resistant to the FLS Chinese Race 7 gray spot [36]; AX-90524893 is close to SS71561457-SS715615158, which is resistant to FLS Race 15 [37]; AX-90430118 is close to Satt114, which is related to FLS Chinese Race 15 [24]; and AX-90524893 is close to Satt335, which is associated with FLS Chinese Race 12 [38]. AX-90508257 on chromosome 16 is close to Gm16:33856661, underlying resistance to Chinese Race 7 [14]. AX-90433524 on chromosome 18 is adjacent to ss715631941, which is resistant to Race 15 [14]. Other loci have not been reported in previous studies, and further investigations of the biological functions of these loci in combination with other experimental methods are needed.

### 4.4. Candidate Gene Screening

An analysis of the colocalization results of the QTL and GWAS revealed that 14 QTNs were within the QTL intervals. In the LD regions of these QTNs, genes related to resistance to FLS were searched on the Phytozom website. Based on the combination of functional annotations, KEGG and GO analysis results, and gene variation analysis, three genes involved in Race 7 in China were identified.

Transcription factors involved in the stress response of plants can regulate gene expression and are key factors for plant stress resistance. *Glyma.06G300100* is a member of the transcription factor MBY family, which plays an important regulatory role in the stress response of plants. The genes in the MBY family are widely present in eukaryotes such as *Arabidopsis thaliana* and maize [39,40]. By forming homologous or heterologous dimers or interacting with other proteins, they can participate in signal transduction pathways such as JA, SA, and ABA, forming a regulatory network for gene expression [41] and thereby improving the stress resistance ability of crops. Therefore, we hypothesized that the *Glyma.06G300100* gene also plays an important role in soybean resistance to FLS Race 7.

*Glyma.06G300600* is a protein with a PAN domain, the core of which is 4–6 cysteine residues, existing in 28,000 proteins of 959 families [42]. It plays an important role in the immunosuppression process in plants. When the PAN domain is mutated, significant differences in MAPK phosphorylation, global transcription reprogramming, downstream signal transduction components, hormone biosynthesis, and resistance to gray mold disease occur [42]. PAN domain-mediated ubiquitination and protein hydrolytic degradation play a role in receptor conversion, thereby inhibiting the defense signal transduction of jasmonic acid and ethylene in plants [43].

Peroxidase is widely present in plants. It is divided into hemin peroxidase and non-hemin peroxidase. The protein encoded by *Glyma.13G172300* is a hemin peroxidase. During the stress response in plants, hemin peroxidase can trigger the defense mechanism of plants against adverse environments as a signaling factor; peroxidase is also a part of the resistance of plants to adverse external environments. When bacteria and fungi invade plants, they can produce toxic substances to resist pathogen infection. Hemin peroxidase is widely involved in the metabolism of reactive oxygen species (ROS), hormone synthesis and decomposition, and defense against the external environment in plants [44]. Therefore, we hypothesized that the *Glyma.13G172300* gene participates in the regulation of soybean resistance to FLS Chinese Race 7.

The families of these three genes (*Glyma.06G300100*, *Glyma.06G300600*, and *Glyma.13G172300*) help regulate the responses of plants to biotic stress. Combined with the positioning results, we hypothesized that these three genes are involved in the regulation of resistance to FLS Race 7 in soybeans.

## 5. Conclusions

This study identified 18 QTNs and 90 QTLs from the two genetic populations. Among them, 14 QTNs were collected from the two populations. Through further investigation, three genes (*Glyma.13G172300*, *Glyma.06G300100*, and *Glyma.06G300600*) proximal to the molecular markers AX-90524088 and AX-90437152 were revealed to potentially be involved in resistance against frogeye leaf spot (FLS) caused by *C. sojina* Race 7 in soybean. These results provide a theoretical basis for understanding the pathogenic genetic mechanism of *C. sojina* and for molecular breeding to improve disease resistance.

Subsequent research should employ functional validation approaches such as overexpression, knockout, and complementation assays to confirm the roles of these three candidate genes. Concurrently, molecular breeding techniques should be utilized to develop elite germplasms with enhanced resistance to *C. sojina* Race 7, while closely linked molecular markers should be designed to accelerate translational applications in disease-resistant molecular breeding.

## Figures and Tables

**Table 1:** Descriptive statistics on the PLAD of soybean FLS in RIL3613 in two environments.

Environment	DN L13 (Average)	Hh 36 (Average)	Average	Standard Deviation	Minimum	Maximum	Skewness	Kurtosis
XY	0.015	0.009	0.007	0.014	0.000	0.114	5.238	35.731
AC	0.038	0.000	0.010	0.016	0.000	0.106	3.371	15.099

**Table 2:** Descriptive statistics on the PLAD of soybean FLS in the GP in the two environments.

Environment	Average	Standard Deviation	Minimum	Maximum	Skewness	Kurtosis
XY	0.031	0.027	0	0.163	1.224	1.398
AC	0.027	0.022	0	0.103	1.092	0.404

**Table 3:** Nine QTLs associated with PLAD were detected in the three environments.

QTL	Eve.	Chromosome	Position	LeftMarker	RightMarker	LOD	PVE (%)	Add
qFLS7-2–2	XY	2	30	36c02048	36c02047	10.3684	0.3497	−0.0454
	AC	2	30	36c02048	36c02047	7.166	1.3612	−0.0301
	Ave	2	30	36c02048	36c02047	6.2352	0.585	−0.0233
qFLS7-2–4	XY	2	92	36c02078	36c02085	9.1225	0.3846	−0.0344
	AC	2	92	36c02078	36c02085	4.3503	2.0034	−0.016
	Ave	2	92	36c02078	36c02085	4.7828	0.6514	−0.0157
qFLS7-9–4	XY	9	75	36c09006	36c09011	11.9232	0.387	−0.0342
	AC	9	75	36c09006	36c09011	9.0694	2.1026	−0.0152
	Ave	9	75	36c09006	36c09011	9.1487	0.6636	−0.0145
qFLS7-9–6	XY	9	129	36c09139	36c09078	12.3221	0.3774	−0.0349
	AC	9	129	36c09139	36c09078	8.3433	2.0573	−0.0155
	Ave	9	129	36c09139	36c09078	7.8439	0.6294	−0.0179
qFLS7-13–2	XY	13	95	36c13012	36c13013	8.1551	0.3872	−0.0342
	AC	13	96	36c13012	36c13013	5.3459	1.9547	−0.0122
	Ave	13	96	36c13012	36c13013	6.9489	0.7106	−0.0167
qFLS7-14–1	XY	14	41	36c14093	36c14072	16.1204	0.3498	−0.0455
	Ave	14	41	36c14093	36c14072	11.992	0.5857	−0.0233
	AC	14	42	36c14093	36c14072	9.0625	1.9965	−0.0164
qFLS7-15–5	XY	15	388	36c15009	36c15010	9.0114	0.3662	−0.0382
	AC	15	388	36c15009	36c15010	11.0052	2.1013	−0.0156
	Ave	15	388	36c15009	36c15010	6.2068	0.6145	−0.0151
qFLS7-17–3	XY	17	89	36c17083	36c17082	13.9472	0.367	−0.038
	AC	17	89	36c17083	36c17082	10.3001	2.2343	−0.0148
	Ave	17	90	36c17083	36c17082	9.6205	0.5853	−0.0233
qFLS7-17–4	XY	17	95	36c17016	36c17017	11.4424	0.3598	−0.0369
	Ave	17	95	36c17016	36c17017	7.0466	0.5835	−0.0204
	AC	17	96	36c17016	36c17017	7.4173	1.3612	−0.0301

**Table 4:** Details of the QTN/QEI associated with the resistance of soybean to the FLS physiological Race 7.

Environ- ment	Marker	Chromo- some	Position (bp)	LOD (Q)	Add	LOD (QE)	Add * env1	Add * env2	r^2^ (%)	*p* Value	Signi- ficance
XY	AX-90327525	3	25,283	6.67	−0.0017				5.08	2.14 × 10^−7^	SIG
XY	AX-90470366	3	44,602,404	7.25	−0.0022				5.32	5.58 × 10^−8^	SIG
XY	AX-90383019	4	12,560,483	5.49	−0.0004				4.53	3.23 × 10^−6^	SUG
XY	AX-90524088	6	48,913,997	10.14	−0.002				7.82	7.27 × 10^−11^	SIG
XY	AX-90443776	9	39,781,488	4.48	−0.0036				2.21	3.30 × 10^−5^	SUG
XY	AX-90430118	13	28,665,638	9.28	0.0026				6.94	5.23 × 10^−10^	SIG
XY	AX-90524893	13	30,790,917	5.65	−0.0003				4.06	2.24 × 10^−6^	SUG
XY	AX-90439650	13	34,989,937	5.63	−0.0011				4.26	2.36 × 10^−6^	SUG
XY	AX-90508257	16	31,840,753	6.26	−0.0046				4.78	5.55 × 10^−7^	SIG
AC	AX-90468600	4	37,488,739	19.83	−0.001				6.80	1.47 × 10^−20^	SIG
AC	AX-90332112	8	7,608,204	16.58	−0.0076				1.42	2.62 × 10^−17^	SIG
AC	AX-90506929	9	34,223,245	4.70	0.0024				1.04	1.98 × 10^−5^	SUG
AC	AX-90463766	14	43,424,209	8.69	0.0006				2.56	2.05 × 10^−9^	SIG
AC	AX-90517572	17	25,347,969	40.20	0.0002				16.5	6.40 × 10^−41^	SIG
AC	AX-90437152	17	36,392,027	82.71	0.0002				49.1	1.96 × 10^−83^	SIG
AC	AX-90433524	18	6,078,523	3.01	−0.0001				0.94	0.001	SUG
Joint	AX-90325055	2	14,798,048	4.88	0.0032				1.35	1.31 × 10^−5^	SUG
Joint	AX-90468600	4	37,488,739	11.13	−0.0001				3.07	7.44 × 10^−12^	SIG
Joint	AX-90319684	5	32,124,284	8.54	0.0011				2.26	2.90 × 10^−9^	SIG
Joint	AX-90517572	17	25,347,969	16.94	0.0015				4.97	1.15 × 10^−17^	SIG
Joint	AX-90437152	17	36,392,027	51.02	−0.0003				17.6	9.49 × 10^−52^	SIG
Joint	AX-90468600	4	37,488,739			5.5474	−0.0007	0.0007	1.51	2.84 × 10^−6^	SUG
Joint	AX-90339041	8	7,384,418			4.2897	−0.0001	0.0001	1.12	5.13 × 10^−5^	SUG
Joint	AX-90506929	9	34,223,245			9.0559	0.0027	−0.0027	2.37	8.80 × 10^−10^	SIG
Joint	AX-90444106	13	1,163,621			4.3602	0.0031	−0.0031	1.14	4.36 × 10^−5^	SUG
Joint	AX-90412520	14	5,629,805			7.4253	−0.0007	0.0007	1.96	3.76 × 10^−8^	SIG
Joint	AX-90517572	17	25,347,969			21.0304	−0.0004	0.0004	6.27	9.34 × 10^−22^	SIG
Joint	AX-90437152	17	36,392,027			52.8551	0.0006	−0.0006	18.4	1.40 × 10^−53^	SIG

NOTE: SIG: significant QTNs based on Bonferroni correction (α = 0.05/m, where m is the number of markers); SUG: suggested QTNs with LOD scores ≥ 3.0 but *p* values > 0.05/m (Bonferroni correction). “add*env1” denotes the additive–environment interaction effect. The asterisk (“*”) specifically signifies an interaction term, representing the combined influence of the additive genetic effect (“add”) and Environment 1/2 (“env1/2”) in statistical modeling.

**Table 5:** The QTN in the GP population colocalized with the QTL of the RIL3613 population.

QTN	Enviro- nment	Chromo- some	Position (bp)	r^2^ (%)	*p* Value	QTL	LeftMarker	RightMarker
AX-90325055	Joint	2	14,798,048	1.346	1.30654 × 10^−5^	qFLS7-2–4	14,075,423	14,807,628
AX-90319684	Joint	5	32,124,284	2.2618	2.89977 × 10^−9^	qFLS7-5–1	5,605,773	34,482,017
AX-90524088	XY	6	48,913,997	7.824	7.27444 × 10^−11^	qFLS7-6–1	45,980,403	50,219,401
AX-90332112	AC	8	7,608,204	1.4221	2.61598 × 10^−17^	qFLS7-8–3	2,730,666	44,849,884
AX-90339041	Joint	8	7,384,418	1.1162	5.1345 × 10^−5^	qFLS7-8–3	2,730,666	44,849,884
AX-90443776	XY	9	39,781,488	2.2125	3.29892 × 10^−5^	qFLS7-9–6	27,334,487	48,719,846
AX-90506929	AC	9	34,223,245	1.0424	1.97962 × 10^−5^	qFLS7-9–6	27,334,487	48,719,846
AX-90506929	Joint	9	34,223,245	2.3736	8.799 × 10^−10^	qFLS7-9–6	27,334,487	48,719,846
AX-90443776	XY	9	39,781,488	2.2125	3.29892 × 10^−5^	qFLS7-9–5	6,963,595	38,517,945
AX-90506929	AC	9	34,223,245	1.0424	1.97962 × 10^−5^	qFLS7-9–5	6,963,595	38,517,945
AX-90443776	XY	9	39,781,488	2.2125	3.29892 × 10^−5^	qFLS7-9–2	22,385,933	43,970,874
AX-90506929	AC	9	34,223,245	1.0424	1.97962 × 10^−5^	qFLS7-9–2	22,385,933	43,970,874
AX-90430118	XY	13	28,665,638	6.937	5.23201 × 10^−10^	qFLS7-13–1	1,712,042	41,981,042
AX-90524893	XY	13	30,790,917	4.056	2.23592 × 10^−6^	qFLS7-13–1	1,712,042	41,981,042
AX-90439650	XY	13	34,989,937	4.2636	2.36049 × 10^−6^	qFLS7-13–1	1,712,042	41,981,042
AX-90463766	AC	14	43,424,209	2.562	2.05101 × 10^−9^	qFLS7-14–1	14,348,437	45,451,538
AX-90517572	Joint	17	25,347,969	4.9732	1.14724 × 10^−17^	qFLS7-17–8	15,210,682	39,468,676
AX-90437152	Joint	17	36,392,027	17.62	9.49252 × 10^−52^	qFLS7-17–8	15,210,682	39,468,676
AX-90433524	AC	18	6,078,523	0.9448	0.00097409	qFLS7-18–1	4,883,393	54,664,500

**Table 6:** Annotation information of candidate genes.

Genes	Location	Annotation
*Glyma.05G128200*	Chr5:32158033..32162343	LRR RECEPTOR-LIKE SERINE/THREONINE-PROTEIN KINASE FLS2
*Glyma.06G300000*	Chr6:48880588..48882272	MYB-LIKE DNA-BINDING PROTEIN MYB
*Glyma.06G300100*	Chr6:48890857..48892413	MYB-LIKE DNA-BINDING PROTEIN MYB
*Glyma.06G300200*	Chr6:48908957..48910448	MYB-LIKE DNA-BINDING PROTEIN MYB
*Glyma.06G300300*	Chr6:48918302..48920771	MYB-LIKE DNA-BINDING PROTEIN MYB
*Glyma.06G300400*	Chr6:48929743..48931574	MYB-LIKE DNA-BINDING PROTEIN MYB
*Glyma.06G300600*	Chr6:48955700..48958858	Protein kinase domain (Pkinase)//S-locus glycoprotein domain (S_locus_glycop)//D-mannose binding lectin (B_lectin)//PAN-like domain (PAN_2)
*Glyma.08G099400*	Chr8:7597974..7602099	CBL-INTERACTING SERINE/THREONINE-PROTEIN KINASE 23
*Glyma.13G172300*	Chr13:28629837..28635052	PEROXIDASE 19
*Glyma.13G172500*	Chr13:28644818..28649188	ZINC FINGER FYVE DOMAIN CONTAINING PROTEIN
*Glyma.13G172900*	Chr13:28697099..28703351	TRANSCRIPTION FACTOR VOZ1
*Glyma.13G194500*	Chr13:30763822..30769223	LEUCINE-RICH REPEAT-CONTAINING PROTEIN
*Glyma.13G239100*	Chr13:34957147..34964627	UBIQUITIN-CONJUGATING ENZYME E2 24-RELATED
*Glyma.13G239300*	Chr13:34970871..34976701	CALLOSE SYNTHASE 11
*Glyma.17G216100*	Chr17:36392667..36394305	ETHYLENE-RESPONSIVE TRANSCRIPTION FACTOR ERF003

**Table 7:** Information on five gene variants with non-synonymous mutations in exon regions.

Gene	Position/bp (Chromosome 6)				Quantity
	49,672,979	49,673,106	49,674,296	49,674,305	
*Glyma.06G300000*	G(Arg)	G(His)	A(Val)	G(Arg)	33
	A(Leu)	G(His)	A(Val)	G(Arg)	90
	A(Leu)	A(Tyr)	G(Ala)	C(Ser)	18
	49,683,236	49,683,328	49,683,551	49,683,641	
*Glyma.06G300100*	G(His)	G(Ala)	G(Gln)	T(Lle)	68
	T(Asn)	A(Val)	C(Glu)	A(Phe)	56
	49,749,059		49,749,773		
*Glyma.06G300600*	C(Gly)		A(Asn)		100
	G(Ala)		C(Glu)		32
	7,696,683				
*Glyma.08G099400*	G(Gly)				152
	T(Cys)				2
	29,825,592				
*Glyma.13G172500*	C(Val)				147
	T(Lle)				1

## Data Availability

Data are contained within the article and Supplementary Materials.

## Supplementary Materials

The following supporting information can be downloaded at https://www.mdpi.com/article/10.3390/plants14131988/s1, Table S1: Quantitative primer sequences. Table S2: ANOVA and heritability of the PLAD of soybean FLS in the RIL3613 population in two environments. Table S3: ANOVA and heritability of the PLAD of soybean FLS in the GP populations in the two environments. Table S4: Information on QTLs related to the resistance of soybean to physiological Race 7 of frogeye leaf spot. Figure S1: Positions of QTLs/QTNs/QEIs related to resistance to soybean frogeye leaf spot Race 7 on chromosomes. Table S5: Information on the variants of 11 genes with mutations in promoter regions.

## Abbreviations

The following abbreviations are used in this manuscript:

Acheng	AC
Xiangyang	XY
Frogeye leaf spot	FLS
Single-seed design method	SSD
Quantitative trait loci	QTL
Quantitative trait nucleotide	QTN
Analysis of variance	ANOVA
Percentage of leaf area diseased	PLAD

## References

[1] Kim H., Newell A.D., Cota-Sieckmeyer R.G., Rupe J.C., Fakhoury A.M., Bluhm B.H. (2013). Mating-type distribution and genetic diversity of *Cercospora sojina* populations on soybean from Arkansas: Evidence for potential sexual reproduction. Phytopathology.

[2] Athow K., Probst A.H. (1952). The inheritance of resistance to frog-eye leaf spot of soybeans. Phytopathology.

[3] Yorinori J.T., Copping L.G., Green M.B., Rees R.T. (1992). Management of foliar fungal diseases in soybean in Brazil. Pest Management in Soybean.

[4] Mian M.A.R., Missaoui A.M., Walker D.R., Phillips D.V., Boerma H.R. (2008). Frogeye leaf spot of soybean: A review and proposed race designations for isolates of *Cercospora sojina* Hara. Crop Sci..

[5] Soares A.P.G., Guillin E.A., Borges L.L., Silva A.C.T.d., Almeida Á.M.R.d., Grijalba P.E., Gottlieb A.M., Bluhm B.H., Oliveira L.O.d. (2015). More Cercospora species infect soybeans across the Americas than meets the eye. PLoS ONE.

[6] Phillips D.V., Boerma H.R. (1981). *Cercospora sojina* race 5: A threat to soybeans in the southeastern United States. Phytopathology.

[7] Xiao G., Fang T., Hou J., Wang W., Sun L. (2024). Advances in *Cercospora sojina* physiological races and inheritance of resistance to soybean frogeye leaf spot. J. Plant Genet. Resour..

[8] Probst A.H., Athow K.L., Laviolette F.A. (1965). Inheritance of resistance to race 2 of *Cercospora sojina* in soybeans. Crop Sci..

[9] Phillips D.V., Boerma H.R. (1982). Two Genes for Resistance to Race 5 of *Cercospora sojina* in Soybeans. Phytopathology.

[10] Yang W., Weaver D.B., Nielsen B.L., Qiu J., Salamini F. (2001). Molecular mapping of a new gene for resistance to frogeye leaf spot of soya bean in ‘Peking’. Plant Breed..

[11] Gu X., Huang S., Zhu Z., Ma Y., Yang X., Yao L., Gao X., Zhang M., Liu W., Qiu L. (2021). Genome-wide association of single nucleotide polymorphism loci and candidate genes for frogeye leaf spot (*Cercospora sojina*) resistance in soybean. BMC Plant Biol..

[12] Liang X., Yang W. (2021). Association analysis of resistance of soybean to *Cercospora sojina* Hara Race 10 based on SSR markers. Chin. J. Oil Crop Sci..

[13] Na C., Miao H., Jiang H., Ma J., Liu H., Lv S., Zhou J., Yang Y., Zhan Y., Teng W. (2023). Genome-wide association analysis of resistance to frogeye leaf spot China race 7 in soybean based on high-throughput sequencing. Theor. Appl. Genet..

[14] McDonald S.C., Buck J., Song Q., Li Z. (2023). Genome-wide association study reveals novel loci and a candidate gene for resistance to frogeye leaf spot (*Cercospora sojina*) in soybean. Mol. Genet. Genom..

[15] Li X., Zhang K., Sun X., Huang S., Wang J., Yang C., Siyal M., Wang C., Guo C., Hu X. (2020). Detection of QTL and QTN and candidate genes for oil content in soybean using a combination of four-way-RIL and germplasm populations. Crop J..

[16] Wang J., Hu B., Huang S., Hu X., Siyal M., Yang C., Zhao H., Yang T., Li H., Hou Y. (2022). SNP-bin linkage analysis and genome-wide association study of plant height in soybean. Crop Pasture Sci..

[17] Yao D., Wang P., Zhang J., Liu Z., Guan S., Liu S., Qu J. (2014). A QTL mapping analysis of main yield traits in soybean. J. South China Agric. Univ..

[18] Zhang K., Liu S., Li W., Liu S., Li X., Fang Y., Zhang J., Wang Y., Xu S., Zhang J. (2018). Identification of QTNs controlling seed protein content in soybean using multi-locus genome-wide association studies. Front. Plant Sci..

[19] Han Z., Ke H., Li X., Peng R., Zhai D., Xu Y., Wu L., Wang W., Cui Y. (2023). Detection of epistasis interaction loci for fiber quality-related trait via 3VmrMLM in upland cotton. Front. Plant Sci..

[20] Li M., Zhang Y., Xiang Y., Liu M., Zhang Y. (2022). IIIVmrMLM: The R and C++ tools associated with 3VmrMLM, a comprehensive GWAS method for dissecting quantitative traits. Mol. Plant.

[21] Liu X., Shang C., Duan P., Yang J., Wang J., Sui D., Chen G., Li X., Li G., Hu S. (2025). The SlWRKY42–SlMYC2 module synergistically enhances tomato saline–alkali tolerance by activating the jasmonic acid signaling and spermidine biosynthesis pathway. J. Integr. Plant Biol..

[22] Mengiste T., Chen X., Salmeron J., Dietrich R. (2003). The *BOTRYTIS SUSCEPTIBLE1* gene encodes an R2R3MYB transcription factor protein that is required for biotic and abiotic stress responses in Arabidopsis. Plant Cell.

[23] Sun M., Na C., Jing Y., Cui Z., Li N., Zhan Y., Teng W., Li Y., Li W., Zhao X. (2022). Genome-wide association analysis and gene mining of resistance to China race 1 of frogeye leaf spot in soybean. Front. Plant Sci..

[24] Pham A.T., Harris D.K., Buck J., Hoskins A., Serrano J., Abdel-Haleem H., Cregan P., Song Q., Boerma H.R., Li Z. (2015). Fine mapping and characterization of candidate genes that control resistance to *Cercospora sojina* K. Hara in two soybean germplasm accessions. PLoS ONE.

[25] Gao Y., Zhu J. (2000). Advance on methodology of QTL mapping for plants. Hereditas.

[26] Yan H., Wang H., Cheng H., Hu Z., Chu S., Zhang G., Yu D. (2015). Detection and fine-mapping of SC7 resistance genes via linkage and association analysis in soybean. J. Integr. Plant Biol..

[27] Li X., Wang P., Zhang K., Liu S., Qi Z., Fang Y., Wang Y., Tian X., Song J., Wang J. (2021). Fine mapping QTL and mining genes for protein content in soybean by the combination of linkage and association analysis. Theor. Appl. Genet..

[28] Sun M., Zhao T., Liu S., Han J., Wang Y., Zhao X., Li Y., Teng W., Zhan Y., Han Y. (2024). QTL Detection of Salt Tolerance at Soybean Seedling Stage Based on Genome-Wide Association Analysis and Linkage Analysis. Plants.

[29] Zhang J., Wang S., Wu X., Han L., Wang Y., Wen Y. (2022). Identification of QTNs, QTN-by-environment interactions and genes for yield-related traits in rice using 3VmrMLM. Front. Plant Sci..

[30] Xiong X., Li J., Su P., Duan H., Sun L., Xu S., Sun Y., Zhao H., Chen X., Ding D. (2023). Genetic dissection of maize (*Zea mays* L.) chlorophyll content using multi-locus genome-wide association studies. BMC Genom..

[31] Niu H., Kuang M., Huang L., Shang H., Yuan Y., Ge Q. (2023). Lint percentage and boll weight QTLs in three excellent upland cotton (*Gossypium hirsutum*): ZR014121, CCRI60, and EZ60. BMC Plant Biol..

[32] Hong H., Li M., Chen Y., Wang H., Wang J., Guo B., Gao H., Ren H., Yuan M., Han Y. (2022). Genome-wide association studies for soybean epicotyl length in two environments using 3VmrMLM. Front. Plant Sci..

[33] He L., Wang H., Sui Y., Miao Y., Jin C., Luo J. (2022). Genome-wide association studies of five free amino acid levels in rice. Front. Plant Sci..

[34] Wei N., Zhang S., Liu Y., Wang J., Wu B., Zhao J., Qiao L., Zheng X., Wang J., Zheng J. (2022). Genome-wide association study of coleoptile length with Shanxi wheat. Front. Plant Sci..

[35] Sonah H., O’Donoughue L., Cober E., Rajcan I., Belzile F. (2015). Identification of loci governing eight agronomic traits using a GBS-GWAS approach and validation by QTL mapping in soya bean. Plant Biotechnol. J..

[36] Hongjiao J., Huiyan Z., Chunyu L., Xiaoyu L., Shumei M., Yang W. (2020). Soybean variety resource resistant to *Cercospora sojina* race 7: Mining of elite alleles. Chin. Agric. Sci. Bull..

[37] McAllister K.R., Lee Y.C., Kantartzi S.K. (2021). QTL mapping for resistance to *Cercospora sojina* in Essex Forrest soybean (*Glycine max* L.) lines. J. Plant Breed. Crop Sci..

[38] Dong Y., Lai W., Ding J., Wang W., Yu Z., Li Z., Yang X., Liu L. (2016). Excavation the resistance locus to *Cercospora sojina* Hara race 12 in soybean by association analysis. Soybean Sci..

[39] Stracke R., Werber M., Weisshaar B. (2001). The R2R3-MYB gene family in Arabidopsis thaliana. Curr. Opin. Plant Biol..

[40] Rabinowicz P.D., Braun E.L., Wolfe A.D., Bowen B., Grotewold E. (1999). Maize R2R3 Myb genes: Sequence analysis reveals amplification in the higher plants. Genetics.

[41] Liu L., Du H., Tang X., Wu Y., Huang Y., Tang Y. (2008). The roles of MYB transcription factors on plant defense responses and its molecular mechanism. Hereditas.

[42] Pal D., De K., Shanks C.M., Feng K., Yates T.B., Morrell-Falvey J., Davidson R.B., Parks J.M., Muchero W. (2022). Core cysteine residues in the Plasminogen-Apple-Nematode (PAN) domain are critical for HGF/c-MET signaling. Commun. Biol..

[43] De K., Pal D., Shanks C.M., Yates T.B., Feng K., Jawdy S.S., Hassan M.M., Prabhakar P.K., Yang J.Y., Chapla D. (2023). The Plasminogen-Apple-Nematode (PAN) domain suppresses JA/ET defense pathways in plants. bioRxiv.

[44] Freitas C.D.T., Costa J.H., Germano T.A., Rocha R.d.O., Ramos M.V., Bezerra L.P. (2024). Class III plant peroxidases: From classification to physiological functions. Int. J. Biol. Macromol..

