# Supplementary figures and images for: Fine Mapping of QTLs/QTNs and Mining of Genes Associated with Race 7 of the Soybean Cercospora sojina by Combining Linkages and GWAS
Source: Plants (Basel). 2025 Jun 29;14(13):1988. doi: 10.3390/plants14131988 (PMC12251640; doi:10.3390/plants14131988)

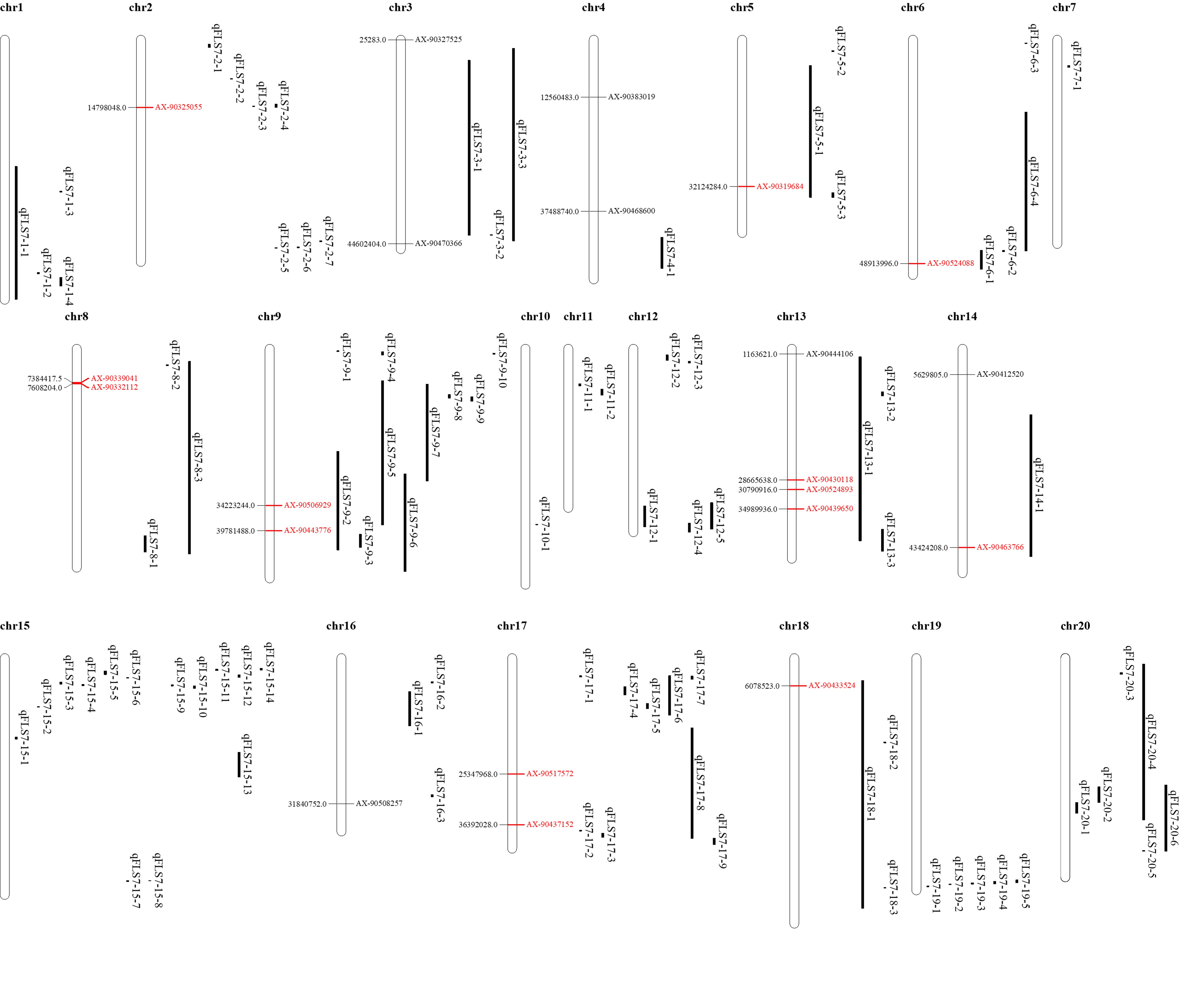

Supplement: Supplementary file 1 [file plants-14-01988-s001.zip › Figure S1 The positions of QTLsQTNsQEIs related to resistance to soybean frogeye leaf spot race 7 on chromosomes.jpg]
