# Supplementary material for: Fine Mapping of QTLs/QTNs and Mining of Genes Associated with Race 7 of the Soybean Cercospora sojina by Combining Linkages and GWAS
Source: Plants (Basel). 2025 Jun 29;14(13):1988. doi: 10.3390/plants14131988 (PMC12251640; doi:10.3390/plants14131988)
Supplement: Supplementary file 1 [file Figure S1 The positions of QTLsQTNsQEIs related to resistance to soybean frogeye leaf spot race 7 on chromosomes.pdf › Figure S1 The positions of QTLsQTNsQEIs related to resistance to soybean frogeye leaf spot race 7 on chromosomes.pdf]

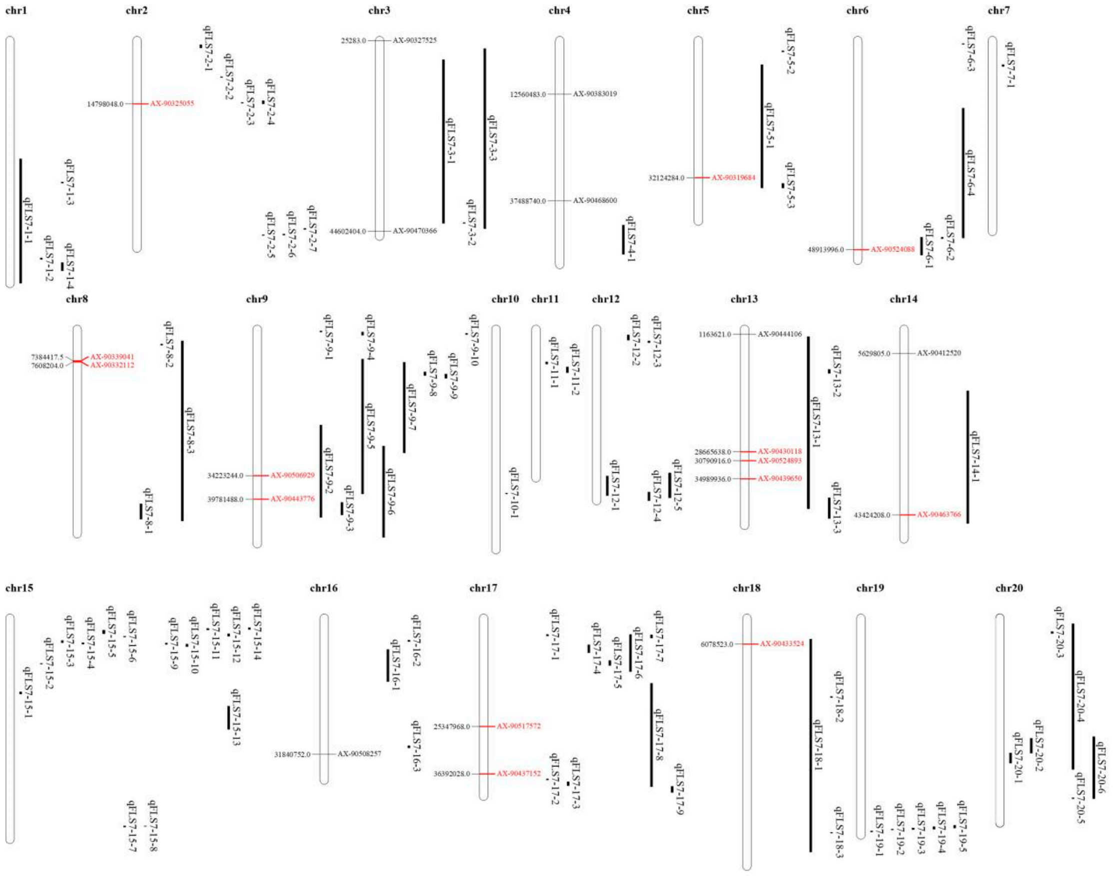

Figure S1 The positions of QTLs/QTNs/QEIs related to resistance to soybean frogeye leaf spot race 7 on chromosomes. Note: The red text indicates the chromosomal positions of 14 QTNs/QEIs co-localized by linkage and association analyses; the black text shows the chromosomal positions of QTNs/QEIs identified solely by association analysis; the black bars represent the chromosomal positions of QTLs mapped via linkage analysis.
